# Decoupling of Temperature and Strain Effects on Optical Fiber-Based Measurements of Thermomechanical Loaded Printed Circuit Board Assemblies

**DOI:** 10.3390/s23208565

**Published:** 2023-10-18

**Authors:** Tiago Maurício Leite, Cláudia Freitas, Roberto Magalhães, Alexandre Ferreira da Silva, José R. Alves, Júlio C. Viana, Isabel Delgado

**Affiliations:** 1 BOSCH Car Multimedia, 4705-820 Braga, Portugal; tiagomauricio.leite@pt.bosch.com (T.M.L.); roberto.magalhaes@pt.bosch.com (R.M.); ricardo.alves3@pt.bosch.com (J.R.A.); isabel.delgado@pt.bosch.com (I.D.); 2 IPC—Institute for Polymers and Composites/LASI, Department of Polymer Engineering, University of Minho, 4800-048 Guimaraes, Portugal; b7833@dep.uminho.pt; 3 CMEMS-UMinho and LABBELS-Associate Laboratory, Department of Industrial Electronics, University of Minho, 4800-048 Guimaraes, Portugal; asilva@dei.uminho.pt

**Keywords:** demodulation, optical fiber sensors, printed circuits, strain measurement, temperature measurement, thermomechanical processes

## Abstract

This study investigated the use of distributed optical fiber sensing to measure temperature and strain during thermomechanical processes in printed circuit board (PCB) manufacturing. An optical fiber (OF) was bonded to a PCB for simultaneous measurement of temperature and strain. Optical frequency-domain reflectometry was used to interrogate the fiber optic sensor. As the optical fiber is sensitive to both temperature and strain, a demodulation technique is required to separate both effects. Several demodulation techniques were compared to find the best one, highlighting their main limitations. The importance of good estimations of the temperature sensitivity coefficient of the OF and the coefficient of thermal expansion of the PCB was highlighted for accurate results. Furthermore, the temperature sensitivity of the bonded OF should not be neglected for accurate estimations of strains. The two-sensor combination model provided the best results, with a 2.3% error of temperature values and expected strain values. Based on this decoupling model, a methodology for measuring strain and temperature variations in PCB thermomechanical processes using a single and simple OF was developed and tested, and then applied to a trial in an industrial environment using a dynamic oven with similar characteristics to those of a reflow oven. This approach allows the measurement of the temperature profile on the PCB during oven travel and its strain state (warpage).

## 1. Introduction

Printed circuit board assemblies (PCBAs) are subjected to high temperatures during their manufacturing process and, later, usually face considerable temperature changes during their operation. PCBs are composed of circuit layers of copper and dielectric material, usually FR4, which is a composite formed with woven-glass-fiber-reinforced epoxy resin [1]. PCBs have several inner and outer conductive layers of diverse geometry and copper distribution. This feature promotes unbalanced loads between layers, which show distinct behaviors under a thermal profile. In order to evaluate the thermal profile of the components during production processes or during their operation, there are currently several methodologies, cataloged as contact or non-contact techniques [2,3,4,5,6]. As non-contact techniques, examples include the infrared camera, liquid crystal thermography and microwave image acquisition. The contact techniques include sensors installed on the surfaces under analysis, such as thermocouples, thermo-resistive sensors, PN junctions and optical fibers (OFs). Thermocouples are the gold standard used in the industry for real-time temperature measurements. Usually, they are installed on key points of the sample via welding or using a high-temperature adhesive, a quick-dry epoxy or Kapton tape dots. Currently, as methods to control the reflow process, the international standards recommend monitoring the temperature of PCBAs at the spots considered critical for the PCB components and solder joints [7]. During the reflow soldering process, and given its heterogeneous composite structure, PCB warpage may occur, which will affect the performance. This thermomechanical process requires adequate online monitoring technology, i.e., temperature and strain measurements at multiple point locations. Currently, this is achieved by placing tens of thermocouples and strain gauges over the PCB at the critical points; this comes with space restrictions, lots of cables with difficult routing and time-consuming mounting operations. Furthermore, the size of these sensors limits their positioning on the PCB (e.g., on the soldering pads). Therefore, OF sensing appears to be a promising technology due to its very small size, the possibility of monitoring temperature and strain in real-time during the thermomechanical processes, the sensors’ multiplexing capabilities and the ease of mounting them over the populated PCB surface, thus overcoming the limitations of the conventional techniques abovementioned. Particularly, distributed OF sensing allows a multitude of monitoring points in a single OF (millimeter range), covering a high number of critical points over the PCB surface (fiber lengths of 1–3 m), at low costs (e.g., as compared to optical fiber Bragg grating (FBG) sensors). In a previous work, OF was investigated for strain measurements on PCBs, specifically using distributed sensing technology [8]. Specifically, OF strain measurements were taken on an in-circuit test (ICT) machine. OF-distributed sensing technology proved to be a suitable alternative for PCB-strain measurement applications.

Due to the dependence of OF sensor measurements on temperature and strain, the simultaneous and distinguishable measurement of these two measures is a difficult task. Several methods have been proposed to measure temperature and strain simultaneously and to decouple both effects on OF measurements based on special OF sensors and techniques [9], including multicore fibers (MFCs) [10]. Barkov et al. [9] investigated a method for temperature–strain discrimination using polarization-Brillouin reflectometry based on two polarization axes of an anisotropic OF (PANDA type). These distinct polarization axes can be considered two independent fibers. The accuracy obtained is dependent upon the calibration of the measurements (temperature and strain sensitivities) and instrument capability (the scanning interval and the signal-to-noise ratio (SNR)). Nevertheless, Brillouin reflectometry provides accurate estimation of the static strain/temperature fields along kilometers with a spatial resolution in the order of centimeters. Gorshkov and Taranov [11] proposed a new OF sensor for simultaneous strain and temperature measurements, adopting a hybrid distributed sensor based on Rayleigh and Raman scatterings. This requires the estimation of the strain and temperature sensitivities of the shift of the Rayleigh scattering and the strain and temperature sensitivities of the normalized anti-Stokes Raman spectra. Very high accuracies on strain and temperature were achieved (1.1 με and 0.04 °C, respectively), but, again, the spatial resolution was high (1–2 m) and measurement times were long (10 min). R. Montanini et al. [12] reported a system composed of two FBGs coupled with different wavelengths that allowed them to simultaneously measure the strain and the temperature. The system was used for monitoring, in real-time, the curing kinetics of glass fiber/epoxy composites. One of the OFs was encapsulated in a smaller-diameter capillary to isolate it from mechanical loads transmitted to the OF when it is incorporated into the epoxy resin. To prevent the loading of the encapsulated fiber caused by the thermal expansion of the capillary material, the OF was cut at one end. A K-type wired thermocouple was placed near the FBG fiber sensor as an independent reference. The wavelength deviations were measured with an FBG interrogation system using a light-emitting diode. Peak wavelength information was extracted from the reflected FBG spectrum through an adjustable single-fiber Fabry–Pérot (FP) filter that scanned the entire 40 nm wavelength range. However, these monitoring modes are located measurements able to measure only a few limited areas. Pedraza et al. [13] studied the application of neural networks for discrimination of temperature and strain in SCF using a phase and polarization analyzer OFDR in a simple approach (single OF, single interrogator). Once trained, the algorithm is capable of distinguishing between strain and temperature readings successfully, with absolute medium errors of 1.9 °C and 60.1 με.

In any case, the use of a single OF sensor to detect temperature and strain remains difficult and further studies are still needed [14]. Other approaches adopted two independent fibers for temperature and strain measurements, respectively. X. Yu et al. [15] used a strain-free short-period FBG for temperature monitoring and low-extrinsic-reflectivity Fabry–Pérot interferometer (EFPI) for strain measurement. The accuracy of the strain sensor after correction was ± 0.6 με with the temperature ranging from 25 °C to 105 °C, with the direct demodulation being improved twice. The temperature demodulation error after calibration was reduced to 1/3 of the direct demodulation, with an accuracy of 1.4 °C. Other studies [16] adopted a method to obtain a distributed decoupling of temperature and strain using two types of Rayleigh backscattered spectra (RBS) and optical frequency domain reflectometry (OFDR). Two types of single mode fiber (SMF) were paired, side by side, as sensory fibers. One was a reduced-cladding (RC) SMF and the other was a standard SMF. This study demonstrated that the measurement errors were 0.31 °C in temperature and 7.97 με in strain, with a measurement range of 50 m and a spatial resolution of 18 cm [16].

There are different methods for decoupling temperature and strain effects in OF measurements, including: the use of special fibers (e.g., MFC, PANDA type), the use of two different OFs (one for strain and other for temperature measurements), the adoption of polarization-Brillouin reflectometry, the adoption of independent interrogation and the use of signal post-processing techniques. Not all have the same spatial resolution, accuracy, ease of operation and costs, and their selection is application-driven. In our case of simultaneous measurements of temperature and strain in the reflow soldering process of PCB, the smallest-diameter sensor is required (as an SCF), with a high spatial resolution (of the order of millimeters), a high speed (order of seconds or less), a high accuracy (same range of conventional thermocouples and electrical resistive strain gauges), a simpler deployment (at workshop floor level) and reduced costs. The OFDR technique for distributed sensing meets all of these requirements. This technique uses swept-wavelength interferometry to interrogate OF sensors [17], measuring the changes in light that is scattered in the OF (Rayleigh scatter). By comparing the scattered light of a sensor with a reference signature measurement that was recorded with the fiber in a known state (OF signature), it is possible to determine the physical state of the fiber at the time of the measurement, which is coupled with the local ambient temperature and strain that, generally, dominate the spectral response or Rayleigh backscatter, besides physical length, and OF refractive index. Local changes in Rayleigh’s scatter cause spectral and temporal shifts in the locally reflected spectrum, which can be scaled to form a distributed sensor. The strain response of the OF occurs due to its physical stretching and the change in the fiber refractive index due to photo-elastic effects. The thermal response of the OF is inherent to the fiber material and to the temperature dependence of its refractive index, n (dn/dT effect represents approximately 95% of the observed variation). The change in the light spectrum spread across the OF in response to strain or temperature variations is similar to a change in the resonance wavelength, ∆λ, or a spectral change, ∆v, of a Bragg grating:

(1) $$\frac{\Delta \lambda }{\lambda }=-\frac{\Delta v}{v}=K_{T}\Delta T+K_{\varepsilon }\varepsilon$$

where λ and v are the mean optical wavelength and frequency, K_T_ and K_ε_ are the temperature and strain calibration constants and ∆T and ε are temperature changes and strain, respectively. The default values for these constants are set at values common for most germanosilicate core fibers, namely: K_T_ = 6.45 × 10^−6^ °C^−1^ and K_ε_ = 0.780. The K_T_ and K_ε_ values are slightly dependent upon the contaminating species and on the concentration in the OF core, but also, to a lesser extent, on the cladding and coating composition.

In this study, the use of distributed OF sensing was investigated using an SCF and OFDR interrogation, to measure temperature and strain variations during the thermomechanical processes of PCB manufacturing. A single OF was used, featuring both strain-free (OF freely moving inside a tube) and strain-sensitive (OF bonded on the PCB) segments for simultaneous temperature and strain measurements. Several temperature–strain demodulation techniques were compared in order to find the best one, suitable for the investigated application. Finally, a methodology for measuring strain and temperature variations in PCB thermomechanical processes was developed and tested. This work proposes the use of OF distributed sensing and OFDR as an advantageous technique for the measurement of the variations in temperature and strain during PCB manufacturing processes, which is of high industrial relevance.

## 2. Optical Fiber Signal Demodulation Models

The simultaneous measurements of temperature and strain with OF remain a major technical challenge, especially when a high accuracy is required in both parameters. Several theoretical formulations for temperature effect compensation on a strain measurement by using an optical signal have been developed, mainly based on a sample preparation method or on an analytical method. In this work, we adopted a point-to-point compensation model with further application of an algebraic method with a combination of the signals from two sensors (temperature, strain) at each spatial point. Three methods are suited for this:

(1) The direct compensation model;
(2) The coefficient of thermal expansion (CTE)-dependent compensation model;
(3) The two-sensor combination model.

The direct compensation model follows a methodology of subtracting the contributions of the two sensors around the same point in space. The sensor bonded to the PCB detects the contributions of both the temperature variation and of the thermo-induced deformation. On the other hand, the strain-free sensor only suffers the contributions of temperature, presenting a smaller magnitude of values. Thus, the measured temperature and strain are calculated from Equations (2) and (3), respectively:

(2) $$T=\Delta \nu_{\mathrm{sf}}\times k_{T}$$

(3) $$\varepsilon =(\Delta \nu_{b}-\Delta \nu_{\mathrm{sf}})\times k_{\varepsilon }$$

where ∆ν_sf_ is the strain-free optical fiber sensor (OFS) signal frequency shift, k_T_ is the temperature sensitivity factor, ∆ν_b_ is the bonded OFS signal frequency shift and k_ε_ is the deformation sensitivity factor. However, this method is quite inaccurate as it neglects the temperature sensitivity of the bonded OF. In fact, a different temperature sensitivity for strain-free and bonded sensors is expected since strain-free sensors are insulated in a PTFE tube and the other ones are bonded to the PCB. 

The CTE-dependent compensation model is also based on a point-to-point compensation technique [18]. The temperature output is calculated using Equation (1), but the deformation output is calculated considering the thermo-optic coefficient of silica optical fiber k_nT_ and the substrate thermal-expansion coefficient α_S_ (Equation (4)) [18]. The main issue in this model is to know the precise value of the substrate CTE (in this case, of the PCB).

(4) $$\varepsilon =\left(\Delta v_{b}\times k_{\varepsilon }\right)-\left[\left(k_{nT}\times \Delta v_{sf}\times k_{\varepsilon }\right)+\left(\Delta v_{sf}\times k_{T}\times \alpha_{S}\right)\right]$$

The two-sensor combination model couples the outputs of both strain-free and bonded sensors to calculate temperature and deformation. The algebraic model (Equation (5)) is based on developed models [19], which were adapted to the present work.

(5) $$\left[\begin{array}{c} {\Delta \upsilon }_{\mathrm{sf}}\\ {\Delta \upsilon }_{b} \end{array}\right]=\left[\begin{array}{cc} k_{\mathrm{sf},T} & k_{\mathrm{sf},\varepsilon }\\ k_{b,T} & k_{b,\varepsilon } \end{array}\right]\left[\begin{array}{c} T\\ \varepsilon \end{array}\right]+\left[\begin{array}{c} \upsilon_{\mathrm{sf},0}\\ \upsilon_{b,0} \end{array}\right]$$

where ∆ν_sf_ is the frequency shift of the strain-free sensor, ∆ν_b_ the frequency shift of the bonded sensor, k_sf,T_ is the strain-free OFS sensitivity to temperature, k_sf,ε_ is the strain-free OFS sensitivity to deformation, k_b,T_ is the bonded OFS sensitivity to temperature, k_b,ε_ is the bonded OFS sensitivity to deformation and ν_sf,0_ and ν_b,0_ are adjustable factors (origin ordinates). Since the strain-free sensor does not sense deformation, k_sf,ε_ = 0. Inverting Equation (5) in terms of temperature and strain, it becomes:

(6) $$\left[\begin{array}{c} T\\ \varepsilon \end{array}\right]={\left[\begin{array}{cc} k_{\mathrm{sf},T} & 0\\ k_{b,T} & k_{b,\varepsilon } \end{array}\right]}^{-1}\left[\begin{array}{c} {\Delta \upsilon }_{\mathrm{sf}}-\upsilon_{\mathrm{sf},0}\\ {\Delta \upsilon }_{b}-\upsilon_{b,0} \end{array}\right]$$

The sensitivity to temperature and strain of the OFS were determined in laboratory calibration tests. The strain and temperature coefficients of a specific OF type could be directly calibrated, recording the spectral deviation for a known applied strain or temperature change. The linear relationships between strain or temperature and the spectral shifts enabled the distributed sensing along any standard single-mode or gradient index multimode fiber, with a millimeter-range spatial resolution over tens of meters of a fiber, and with strain and temperature resolutions better than 1 με and 0.1 °C, respectively [20]. In this study, these demodulation techniques were compared to find the one giving the best measurement accuracy.

## 3. Materials and Methods

### 3.1. Materials

A double layer bare PCB (FR4) with solder mask was used as the testing sample. A polyimide-coated single mode OF, with a LC/APC connector, was used. The OF layout considered the best practices of optical sensing, such as the minimum curvature (>5 mm) or sharp edges that could induce noise in the optical signal, or in extreme cases, break the fiber. The OF sensor fingerprint must be acquired when the sample construction is fully completed for better sensor operation. An M-Bond 610 (VISHAY^®^, Shelton, CT, USA) adhesive was employed to bond an OF segment to the PCB. PI-32 (Kyowa, Japan) was used for fixing the PTFE sleeve. This PTFE sleeve was used to protect few segments of the OF from deformations induced by the thermal expansion of the substrate (strain-free O segments). We used a 24 AWG PTFE sleeve of 0.56 mm internal diameter. A 0.15 mm thick aluminum tape was used around the PTFE sleeve to protect the reference fiber from signal noise induced by convection currents, and to ensure thermal homogeneity at the measurement location.

### 3.2. Testing Sample Preparation

**Figure 1:**
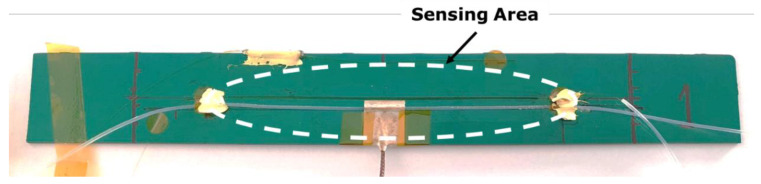
PCB test specimen with dimensions of 200 × 25 × 1.6 mm and with 2 OF segments.

The test specimens were PCB with 200 × 25 mm × 1.6 mm dimensions, as shown in Figure 1. A single OF was used with a segment bonded to the PCB and another freely inserted in the PTFE sleeve. Calibrated thermocouples were used for temperature reference. A K-type thermocouple (RS Components, Northants, UK) was mounted on the sample, directly over the PCB substrate, and fixed with standard aluminum tape (KIC, San Diego, CA, USA) with a thickness of 0.15 mm to ensure thermal homogeneity.

### 3.3. PCB Testing for Calibration

In order to characterize the contribution of the thermo-induced deformation on the OF response, 3-point flexural tests were carried out at room temperature up to 250 °C, in discrete levels, using the universal mechanical testing equipment INSTRON 3300 series, which is capable of acquiring displacements and forces. Five levels of oven stabilization (60, 120, 150, 200 and 250 °C) beyond the initial temperature of 23 °C were chosen, in a total of six temperatures levels. The PCB specimen was loaded perpendicularly to its surface with a 2 mm deflection at its midspan at each level of temperature, at a velocity of 2 mm/min.

### 3.4. PCB Testing in a Dynamic Oven

**Figure 2:**
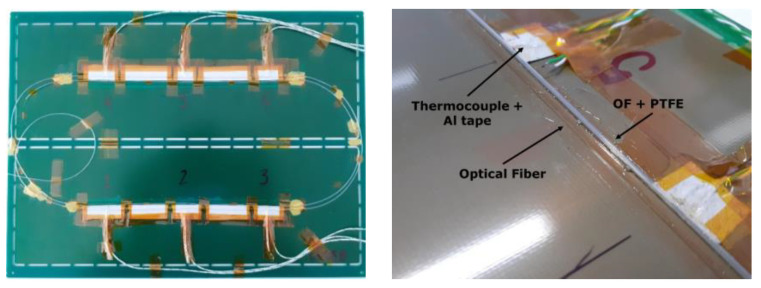
Instrumented PCB with 6 thermocouples and a single OF with segments bonded to the PCB and inside a bonded PTFE sleeve. The numbers are the references of the six strain gauges used at different positions, three in each side of the OF segments.

An instrumented PCB with dimensions of 300 × 210 × 1.6 mm was tested in a dynamic oven. A single OF with Ø155 µm diameter, with a polyimide coating and with an LC/APC connector and a metallic terminal (LUNAinc^®^, Roanoke, VA, USA), was used. The OF was 6 m long with different segments: free OF with generous radius (at the beginning, between segments and at the end of the OF); OF bonded to the PCB (100 mm segments) for strain measurements; and OF free inside a PTFE sleeve 100 mm long bonded to the PCB, for temperature sensing. Six thermocouples were placed in selected locations, three in each top and bottom segment, as depicted in Figure 2.

**Figure 3:**
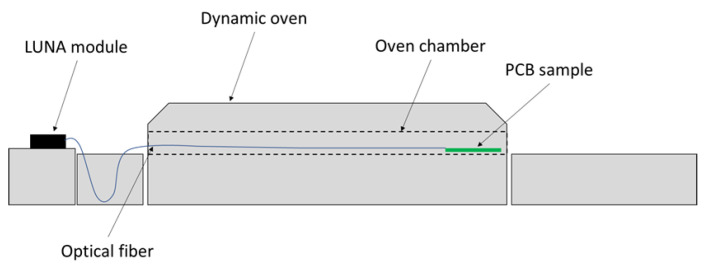
Dynamic oven sample.

The OF was connected to the high-definition optical fiber detection system (HD-FOS) ODiSI-B from LUNAinc^®^ equipped with a class 1 laser and one acquisition channel. This OF interrogator adopts an OFDR technique. The distributed sensing approach allowed measuring temperature and strain at each 2.6 mm over the OF length, with a frequency of 100 Hz and at an acquisition rate of 1 Hz. The OF interrogator was placed outside a dynamic oven, as shown in Figure 3. The OF measures the temperature and strain while the PCB sample progresses on a conveyor inside the oven chamber (left to right).

The dynamic oven is 6 m long and 0.233 m wide, and the conveyor velocity was 0.100 m/min. No practical issues arose regarding the samples’ transportation and its influence on the OF signal. Special care was taken when constructing the connecting portion of the OF, so it would be tension-free and allow the progression of the sample inside the oven at a steady velocity.

## 4. Results and Discussion

### 4.1. Simultaneous Temperature and Strain Tests

**Figure 4:**
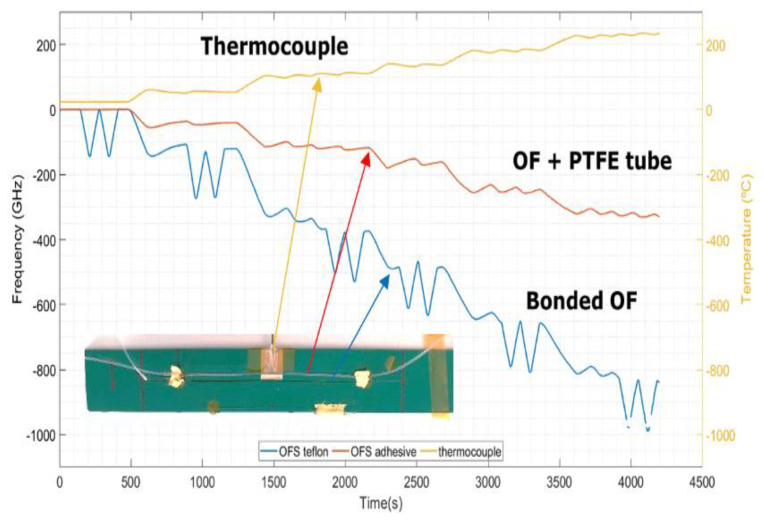
Results of the 3−point flexural tests.

Figure 4 shows the simultaneous measurements of the temperature and strain from the OF under the three-point bending test. As one can observe, the OF dedicated to temperature measurements does not respond to the deformations induced on the sample. In fact, its evolution is alike the thermocouple response. It can, therefore, be assumed that the entire output of this sensor is only due to the temperature variations in the sample.

**Figure 5:**
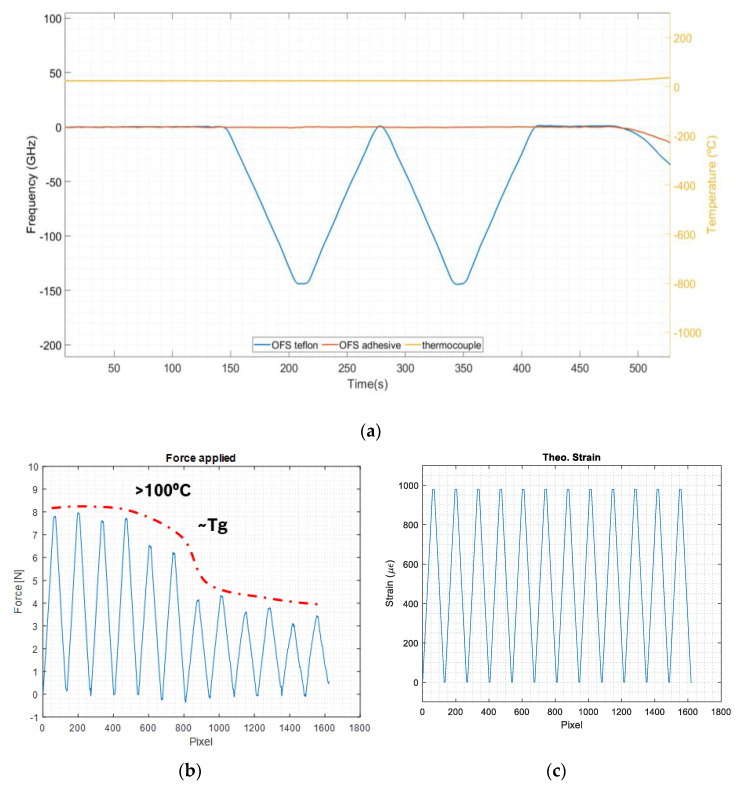
Results of the 3−point flexural test: (**a**) two loading cycles of 2 mm of deflection at constant temperature (c.a. 23 °C); (**b**) force variation over the deformation cycles (the curve in red represents the temperature variations); (**c**) imposed strain variations over the deformation cycles.

The output of the OF bonded to the PCB substrate shows two distinct behaviors: (i) the frequency variations in the optical signal as a function of the temperature, following the shape of the curves of the PTFE + OF sensor and of the thermocouple, and (ii) the frequency variations in the optical signal at each temperature level, i.e., the response to the mechanically induced deformations. It appears that the sensor response to pure strain is approximately constant at each temperature increment (2 mm bending ≈ −150 GHz). However, the force required to apply the 2 mm deflection decreases with the increment of the temperature, displaying a step down from 100 °C, and a stabilization from 150 °C onward. This decrease in the force is, most likely, due to an increased flexibility of the substrate material (FR4) after exceeding its glass transition temperature T_g_ (the T_g_ of FR4 is c.a. 135 °C). Figure 5 presents this data interpretation and summarizes the sensor response to the induced mechanical deformation at each temperature level.

The OF sensor response to temperature can be obtained when calibrating these results with the reference thermocouple data. This includes the response of the OF to the thermomechanical deformation, and its expansion with temperature, among other effects of the OF and of the used adhesive. On the other hand, by considering the portion of the signal under induced strain (at a constant temperature), the sensor response only under strain can be obtained. In this way, temperature and strain calibrations can be performed separately, as shown in the next section.

### 4.2. Calibration Results

#### 4.2.1. Temperature Calibration, k_sf,T_

**Figure 6:**
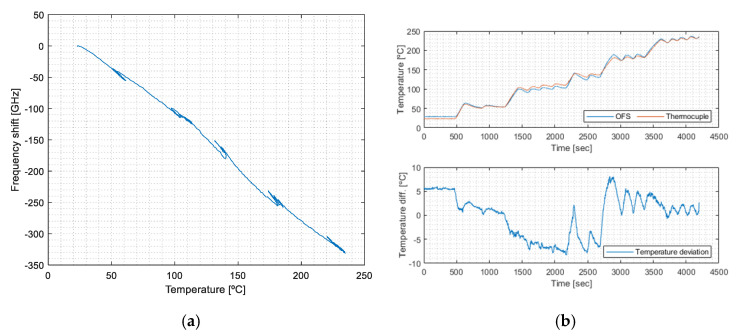
(**a**) Temperature calibration, and (**b**) temperature deviation from reference thermocouple measurements.

Temperature calibration relates the strain-free sensor’s output to the reference thermocouple data (solid and dotted lines in Figure 4), as presented in Figure 6a. Figure 6b shows the temperature deviations from the reference thermocouple measurements.

The temperature calibration coefficient is obtained from the slope of the data of Figure 6a (Equation (7)), with $k_{\mathrm{sf},T}=-1.61\;\mathrm{GHz}\cdot {℃}^{-1}$.

(7) $$\Delta v_{\mathrm{sf}}=-1.61\;\mathrm{GHz}\cdot {℃}^{-1}\times T+v_{\mathrm{sf},0}$$

#### 4.2.2. Thermo-Induced Deformation Calibration, k_b,T_

**Figure 7:**
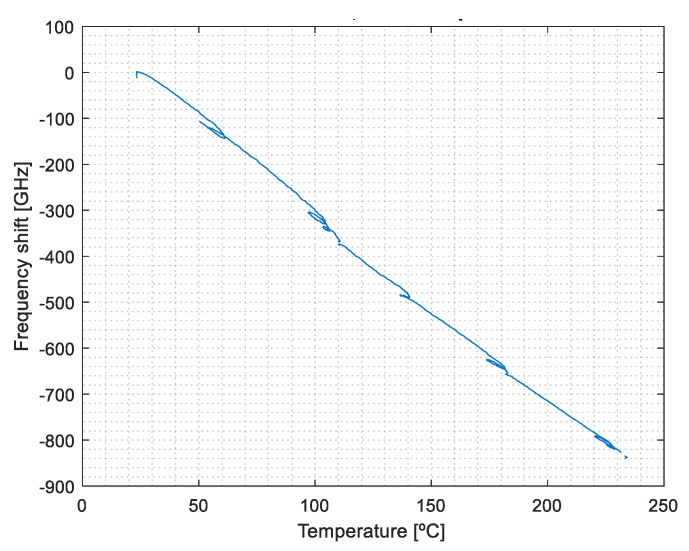
Calibration of the thermal response of the bonded OFS.

The thermo-induced deformation relates the signal of the bonded OF sensor to the reference thermocouple data, except when mechanically induced deformation is applied. The influence of the OFS signal (bonded) under temperature can be determined by correlating the dashed blue curve trend with the thermocouple data (Figure 4), ignoring the mechanically deformed segments. Its output relates to the temperature sensitivity and the subsequent thermo-induced deformation sensitivity of the bonded OF segment. The results are shown in Figure 7.

This calibration is obtained from the trendline adjustment of the data of Figure 7 (Equation (8)), with $k_{b,T}=-4.06\;\mathrm{GHz}\cdot {℃}^{-1}$. This value is different from that of the strain-free calibration previously performed (Equation (7)), showing that k_sf,T_ < k_b,T_, i.e., the bonded OF is more sensitive to the temperature than the strain-free one. In fact, k_bT_ also includes the effect of the thermal strain originating from the difference in the CTE between the OF and the PCB [21].

(8) $$\Delta v_{b}=-4.06\;\mathrm{GHz}\cdot {℃}^{-1}\times T+v_{b,0}$$

#### 4.2.3. Mechanically Induced Deformation Calibration, k_b,_ε

**Figure 8:**
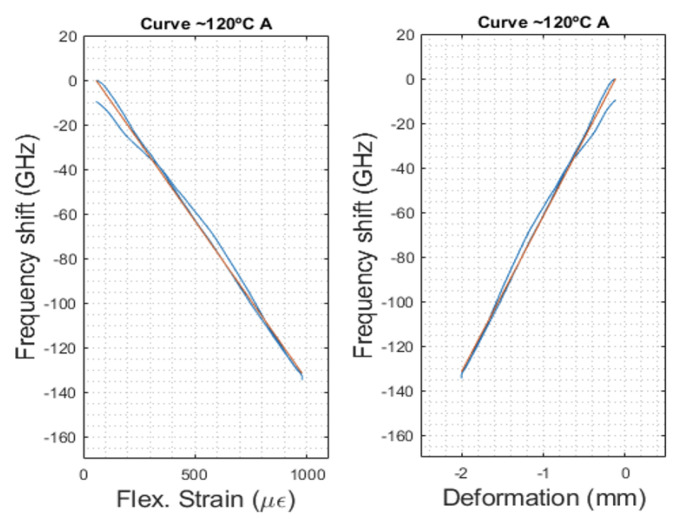
Calibration of the mechanically induced deformation. The dotted red lines represent the linear trendlinex of the experimental data (blue curves).

In the calibration of the mechanically induced deformation, only the OFS signals during the stable temperature levels (∆T ≈ 0 °C) were analyzed. To determine the influence of the OFS signal under deformation alone, only the portions of the lower line curve of Figure 4, with purely mechanical deformation (triangular shaped responses), were considered and related to the data acquired by using the mechanical test machine. As an example, the calibration curves for a constant temperature of 120 °C are shown in Figure 8.

From the equations of the trendlines of each deformation cycle, an average calibration value was calculated, as is shown in Equation (9), with $k_{b,\varepsilon }=-0.158\;\mathrm{GHz}\cdot {\mu \varepsilon }^{-1}.$ The response of the bonded sensor (frequency shift) to the mechanically induced deformation is identical, regardless of the temperature level, i.e., k_b,ε_ displays a stable value throughout the entire test.

(9) $$\Delta v_{b}=-0.158\;\mathrm{GHz}\cdot {\mu \varepsilon }^{-1}\times \varepsilon +v_{b,0}$$

In summary, the demodulation model is governed by the following set of equations (Equation (10)), with the temperature and strain sensitivity coefficients in $\mathrm{GHz}\cdot {℃}^{-1}$ and $\mathrm{GHz}\cdot {\mu \varepsilon }^{-1}$, respectively:

(10) $$\left[\begin{array}{c} T\\ \varepsilon \end{array}\right]=\frac{1}{\det}\left[\begin{array}{cc} -0.158 & 0\\ 4.06 & -1.61 \end{array}\right]\left[\begin{array}{c} \Delta v_{\mathrm{sf}}-v_{\mathrm{sf},0}\\ \Delta_{\mathrm{vb}}-v_{b,0} \end{array}\right]$$

### 4.3. Calibration Method Comparison

**Figure 9:**
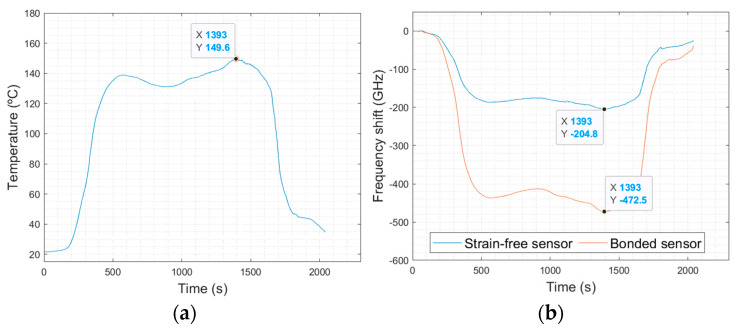
(**a**) Oven reference temperature profile measured usinh a K−type thermocouple, and (**b**) OFS response to the applied thermal cycle.

The three abovementioned models were compared for a better understanding of their accuracies and drawbacks, taking as an example the temperature profile used in the trials in a dynamic oven, in an industrial environment. Figure 9 shows the temperature profile and OFS measurements. Picking up the assigned maximum temperature point (t = 1393 s; T = 149.6 °C), the temperature and deformation from the signal acquired at the OFS can be calculated using the three abovementioned methods. The following values for the frequency of the strain-free sensor, v_sf_ = −204.8 GHz, and for the bonded sensor, v_sb_ = −472.5 GHz, were considered. Note that these values mean that the frequency shift of a bonded OF is much higher than a strain-free OF, as would be expected due to the superposition of strain and temperature effects in the former. Furthermore, both effects appear to have similar influences, as v_sb_/v_sf_ = 2.3. Also, the following values of k_T_ = −0.645 °C·GHz^−1^ for temperature sensitivity and k_ε_= −6.05 με·GHz^−1^ for strain sensitivity were used in the first two models. The thermo-optical coefficient of the OF is 0.95 and the CTE of the PCB substrate (in *X*/*Y* directions) is in the range of 15.3 to 17.7 ppm/°C (determined in a thermomechanical analysis experiment).

Table 1 compares the calculated temperatures and strain values from the three models. Considering these results, the direct compensation model gives a strain component with non-convincing values (of +1620 με!). Note that the sign of the strain values (negative strain vs. positive strain) represents the strain direction. The temperature is underestimated by −17.6 °C. These differences point out the need for a better estimation of k_T_ and the high inaccuracy of neglecting the temperature sensitivity of the bonded OF.

The CTE-dependent model seemed to be better fitted for both components, although with a great discrepancy in the temperature value (giving the same value as the direct compensation method) and inaccuracy in the strain value (now giving a negative value, i.e., a compression sate). This latter error is due to the high model dependency on the precise CTE value of the PCB, which is a value difficult to obtain and that is highly dependent on the PCB layout (e.g., no. of layers, Cu content). The two-sensor combination model gives the best results. The local temperature is overestimated by only −3.4 °C (an error of 2.3%), and an expectant strain value in the order of −186 με (considering the PCB specimen dimensions, this corresponds to an estimated deflection of 0.77 mm).

### 4.4. Dynamic Oven Testing

**Figure 10:**
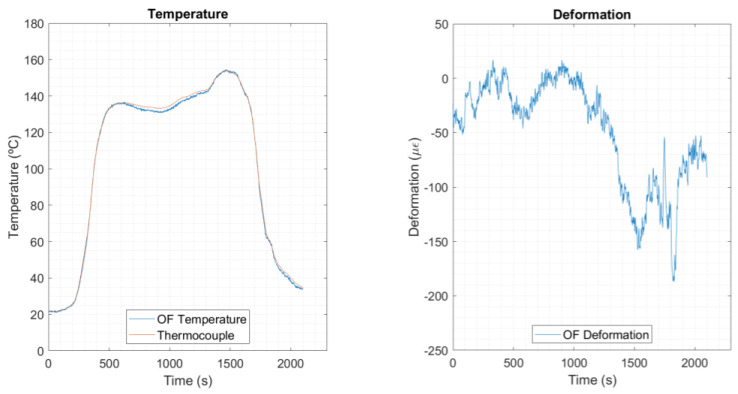
Temperature and deformation results for spot 4.

The demodulation model was applied to a trial in an industrial environment, using a dynamic oven with similar characteristics as a reflow oven. Six sensing spots were pre-selected over the PCB for detailed analysis, each one accompanied by a reference thermocouple of type K. The results after applying the selected demodulation model (two-sensor combination model) are presented in Figure 10. The model was run in a post-processing MATLAB routine (MathWorks, Natick, MA, USA), version R2019Bb.

Looking firstly to the temperature component, one can see that the OF temperature curve follows the profile of the reference thermocouple curve. Thus, it can be ensured that the temperature measurements, except for the error margins inherent to its calibration, present very satisfactory results.

Considering the deformation curve, an evident step down can be seen near t = 1300 s (≈140 °C, close to T_g_ of the PCB FR4 material). Until then, the deformation remains fluctuating in a range from 10 to −50 με. Then, it grows negatively, up to a maximum point of −190 με, revealing warpage of the PCB. It is assumed that this result is a local strain value, matching the deformation in the OF direction, with the overall warpage showing an additional contribution in the z-axis (off-plane of the PCB surface).

## 5. Conclusions

In this study, distributed optical fiber sensing, based on the OFDR technique, was used to measure in (quasi) real-time the temperature and the strain variations during thermomechanical processes of PCB fabrication. Due to the simultaneous temperature and strain sensitivities of OF sensing measurements, several demodulation techniques were compared. The simple direct compensation models showed nonsense results and were disregarded. The CTE-dependent model showed imprecise results of both temperature and strain values, which were related to the precision of the input values, namely k_T_ of the OF and the CTE of the PCB. The two-sensor combination model gave the best results, with a temperature error in the order of 2.3% and realistic strain values (not evaluated by other methods in this work). The comparison of these demodulation models allowed us to draw relevant conclusions: (a) it is important to have good estimations of the temperature sensitivity coefficient for accurate temperature results, as would be expected; (b) precise values of the CTE of the OF and PCB are required for accurate strain calculations, but these are difficult to obtain; (c) the temperature sensitivity of the bonded OF should not be neglected for good estimations of strain; and (d) the two-sensor combination model is the most suitable model for decoupling temperature and strain effects on OF distributed sensing measurements. Based on this demodulation model, a methodology for measuring strain and temperature variations in PCB thermomechanical processes was developed based on a single OF with bonded segments to the PCB for local measurements of the strain, and free OF segments freely moving inside a PTFE sleeve bonded to the PCB for local measurements of the temperature. The two-sensor combination demodulation model was implemented in a MATLAB routine, for post-processing of OF measurements, and applied to an experimental trial in an industrial dynamic oven with similar characteristics to a reflow oven. This approach allowed the successful measurements of the temperature profile on the PCB during oven travelling and of its deformation during this heating cycle. In future, the two-sensor combination model will be improved for faster calculation outputs, as required for real-time measurements. This approach will be deployed and validated in a reflow oven under industrial PCB production for the simultaneous measurements of the temperature and thermo-induced strain (warpage) profiles, aiming at achieving high-quality products.

## Figures and Tables

**Table 1:** Demodulation model comparison.

Direct compensation model		T=132 °C
	$\varepsilon =(\Delta \nu_{b}-\Delta \nu_{sf})\times k_{\varepsilon }$	ε=1620 με
CTE-dependent compensation model	$T=\Delta \nu_{sf}\times k_{T}$	T=132 °C
	$\varepsilon =\left(\Delta \nu_{b}\times k_{\varepsilon }\right)-\left(\left(k_{nT}\times \Delta \nu_{sf}\times k_{\varepsilon }\right)+\left(\Delta \nu_{sf}\times k_{T}\times \alpha_{S}\right)\right)$	$\varepsilon =\left[-656;-339\right]\;\mu \varepsilon$
Two-sensor combination model	$T=\frac{1}{det}\times \left[k_{b,\varepsilon }\;\left(\Delta \upsilon_{sf}-\upsilon_{sf,0}\right)\right]$	T=153 °C
	$\varepsilon =\frac{1}{det}\times \left[k_{b,T}\;\left(\Delta \upsilon_{sf}-\upsilon_{sf,0}\right)+k_{sf,T}\left(\Delta \upsilon_{b}-\upsilon_{b,0.}\right)\right]$	ε=−186 με

## Data Availability

Not applicable.
